# Physiotherapists’ Perceptions of the Influence of Their Health Behaviours on Their Advice to Patients

**DOI:** 10.7759/cureus.35396

**Published:** 2023-02-24

**Authors:** Mariana F Sousa, Fiona Moor

**Affiliations:** 1 Musculoskeletal Physiotherapy, Ascenti Physio Limited, Leicester, GBR; 2 Health and Life Sciences, Coventry University, Coventry, GBR

**Keywords:** health promotion, physiotherapists’ counselling, physiotherapists’ health, lifestyle behaviours, qualitative study

## Abstract

Background

Healthcare professionals (HCPs) lifestyle behaviours can impact their health promotion counselling to patients. However, there is a lack of qualitative studies to understand the physiotherapists' perceptions of the influence of their lifestyle behaviours on their patient's advice.

Aim

This research aims to explore the physiotherapists' perceptions of the influence of their health behaviours on their counselling of patients.

Method

This research was a qualitative study based on the interpretivism paradigm. 15 virtual semi-structured interviews were performed with physiotherapists working for a private company in the United Kingdom (UK). Thematic analysis was used to create four themes and ten sub-themes.

Results

13 out of 15 participants stated that their lifestyle impacts their counselling, while for the other two, their counselling was based on their knowledge. Some drivers for health promotion included role modelling, having some knowledge concerning certain lifestyle behaviours and understanding their importance in health. Barriers to health promotion included lack of time and knowledge, some confusion if discussing some of these behaviours included in their scope of practice and perception of patients’ reactions to certain questions and their relevance to the musculoskeletal (MSK) condition their patients were experiencing. Some strategies proposed to improve their health promotion skills included improvements in communication skills, discussions and sharing of evidence between peers and informative leaflets to distribute to patients.

Conclusion

In this study, 13 out of 15 participants believed their lifestyle impacts their counselling to patients. Despite this, multiple barriers to health promotion were identified. This study highlights the need for training physiotherapists about certain lifestyle behaviours, including smoking cessation, alcohol misuse and basic nutrition counselling which may improve their health promotion messages, potentially leading to patient behaviour change which ultimately may have a positive public health impact.

## Introduction

In 2016, around 71% of deaths (40.5 million individuals) globally were attributed to non-communicable diseases (NCDs) [1]. Of these, 80% (32.2 million individuals) were due to cancers, cardiovascular diseases, chronic respiratory diseases and diabetes, which are the most common NCDs [1,2]. In the same year, in England, 89% of its mortality was due to NCDs (533,100 individuals) and 11% of these deaths were likely premature [2]. These health conditions, which at times can be prevented, are associated with behavioural risk factors such as poor nutrition, lower physical activity (PA) levels and alcohol and tobacco intake [3].

To help individuals and their communities to reduce their risk of disease, the “Making Every Contact Count” (MECC) was implemented in the United Kingdom (UK) [4]. MECC helps the National Health Service (NHS), relevant agencies, local authorities and healthcare professionals (HCPs), to promote behaviour change within the population to improve people’s mental and physical health as well as their well-being and to help HCPs signposting patients to local services [4]. Another aim of this program is to assist individuals to make better decisions about their health, by developing their behaviour change skills [4]. Physiotherapists, who, to improve an injury or disability, provide non-invasive treatment such as prescribing therapeutic exercise and education and have relatively frequent and prolonged contact with patients, are also HCPs with qualifications to educate people to improve their lifestyle [5,6]. An initial evaluation of the MECC showed that it has the potential to be a simple and low-cost resource for HCPs to use across varied health issues [7]. However, evidence showed that HCPs, including nurses, physicians and physiotherapists, reported some barriers when discussing lifestyle recommendations such as nutrition, PA levels, and tobacco and alcohol intake with their patients [8,9]. Some of these barriers included inadequate monetary compensation, lack of knowledge and confidence in advising patients, lack of time and patient non-compliance with the advice given [8,9].

A representative study of the English population, published by Kyle et al. in 2017 [10], indicated a high obesity prevalence in HCPs, such as nurses (25.12%) and other HCPs (14.39%), which included doctors and physiotherapists. This has important implications as it can impact the delivery of health promotion messages from the HCPs to the patients [10]. Black et al. [11] showed that patients believe physiotherapists should be role models for certain health behaviours such as keeping a healthy weight. HCPs’ counselling is strongly associated with their health practices and some of them reported difficulty in advising patients about a health behaviour they were also finding hard to follow [12,13]. Providing strategies to HCPs so they can improve their health behaviours may not only have benefits for their own health but also for their patient’s health [14] and therefore impact the health of the wider public.

There is a lack of evidence that explores the HCPs’ perceptions, especially physiotherapists, of the influence of their lifestyle behaviours on their patients' advice concerning the adoption of healthy behaviours and most evidence available about this topic was performed in physicians and nurses [9,13]. To fill this literature gap, this research aims to explore the physiotherapists' perceptions of the influence of their health behaviours on their counselling of patients.

## Materials and methods

Design

The study design was based on the interpretivism paradigm [15]. It aimed to understand the physiotherapists' perceptions of the influence of their health behaviours on their counselling to patients. To promote a natural and genuine interaction between the participants and the main researcher, virtual semi-structured interviews were the selected design for this project [15].

Participants

To determine the most appropriate sample size, the researchers needed to do a balance between a small sample that would allow them to cope with the amount of data for analysis during a specific timeline to complete the project and a sample that would allow enough in-depth information to answer the research question. The researchers agreed on 15 interviews. The sample size included 15 MSK physiotherapists from a UK private company. To participate in this research, the physiotherapists needed to be registered and have direct contact with patients virtually or in a clinical setting. Those who did not have direct contact with patients or had been on a career break longer than 12 months were excluded.

Ethical considerations

All participants provided written informed consent before participating in this study. It was approved by the private company through its own internal ethics board as well as the Coventry University Ethical Approval (CUEA). The principles of respect for autonomy and confidentiality, beneficence, dignity and justice were taken into consideration to guide the main researcher's actions when collecting data from the participants [16].

Recruitment

After ethical approval, an advertisement post was published in the private company’s weekly newsletter and in some of the company’s Microsoft Team (MT) groups, to recruit volunteers, with information that included the study title, the sample size needed, the study design and the main researcher's contacts. Those who contacted the main researcher and met the inclusion criteria received the “Participant Informative Sheet” (PIS) and the “Consent form” by email and those who agreed to take part in this project received details to arrange an interview date and time. Participant 12 decided not to take part in the interview after receiving the PIS. Due to a numbering error, there was no participant 8. The first 15 participants who met the inclusion criteria and demonstrated interest in participating in this research project were selected for a virtual interview and were anonymised by number allocation.

Data collection

Taking the research question into consideration the researchers discussed and agreed on what specific and probing questions the participants would be asked. Some questions were based on a topic guide and two questionnaires from existing qualitative and quantitative literature [6,17,18]. A pilot interview was undertaken prior to the start of the study to ensure the data collection methods and the structured interview questions were appropriate [15]. Following a review of the pilot, some closed questions were replaced with more open ones. 15 semi-structured interviews were performed through MT between November and December 2021. Each interview was recorded with the participants' permission. An initial transcription was performed by MT. The main researcher listened to the recordings and made the necessary adjustments in each transcription. All of them were reviewed twice to improve their accuracy. During each interview, the main researcher did not provide her personal views concerning the topics discussed to prevent influencing the information given by each participant [15]. However, rephrasing some questions as well as prompting was necessary for some situations where participants did not understand the open questions and/or only provided a brief answer. Although leading questions were avoided during the interviews, these were needed on rare occasions when participants still misunderstood the rephrased question.

Data analysis

The thematic analysis was based on the phases described by Braun and Clarke [19]. To improve the study’s credibility and dependability the themes, sub-themes and quotations were reviewed by the second researcher [16]. Reflexivity was also considered in our study to allow the main researcher to understand the level of influence she might have over the participants [15]. A reflective diary explaining how the research project was going, describing incidents that occurred during the interviews and data analysis was written as a strategy to overcome the researcher’s positionality [15].

## Results

Participants’ information can be found in Tables 1, 2. The interviews' length lasted between 32 and 86 minutes. Table 1 describes some of the participants' demographic information and Table 2 describes participants' lifestyle behaviours.

**Table 1:** Participants' details

Individual Identifiable Number	Age	Gender	Experience (y – years; m-months)	Country of origin	Currently living location
1	38	Male	6y	England	London
2	34	Female	9y	Portugal	Peterborough
3	29	Female	7y	England	Yorkshire
4	24	Female	3y	England	Coventry
5	33	Female	9y	Lithuania	Lincolnshire
6	27	Male	11m	Greece	Bedford
7	27	Female	4.5y	England	Cardiff
9	54	Female	32y	Greece	Coventry
10	27	Female	2y	India	Derby
11	30	Female	5y	India	Northampton
13	30	Female	7y	India	Nottingham
14	30	Female	8y	England	Bradford
15	36	Female	9y	Iran	Nottingham
16	31	Male	10y	England	Cambridgeshire
17	34	Male	6.5y	Greece	Birmingham

**Table 2:** Participants' lifestyle behaviours

Weight	Fruit and vegetable intake	Physical activity	Smoking	Alcohol intake
Six were happy with their weight	Only three follow the “5 a day” recommendation daily	Only eight participants follow the recommended guidelines for PA	None currently smoke	Ten participants drink alcohol, but four of these rarely consume it
Four reported being overweight	Another three follow it most of the time	Main barrier: lack of time	Participant 2 stopped smoking “… a few years ago”	Main reasons for alcohol consumption: social aspect surrounding its intake and liking its taste
Three wanted to lose weight	Main barriers: lack of time and not wanting to eat these types of food.		Main reason for not smoking: knowledge of its harmful health consequences	Five participants do not drink any alcoholic beverage
One was not happy with the weight				Main reasons for not consuming alcohol: not liking its taste and religious decision
One wanted to gain weight				

After thematic analysis, four themes and ten sub-themes emerged from the data. Participants’ quotations were used to illustrate the themes.

Drivers to health promotion

Being a Role Model

Some physiotherapists believed that they needed to set a good example when counselling patients about healthy lifestyles.

*“Yeah, you know you have to set a good example. Ahm… As a health professional I can't be giving out information about being healthy and I I don't do it myself so, you know, just by leading by example, really...”* (Participant 1).

However, one participant believed that patients expect them to be role models and if they believe they are not, then this could create some barriers when discussing health behaviours with them.

*“… I think the way you are, the way you look ahh ah it has an impact on on that as well and I don't think, I don't know how… How much a patient would trusts something that I would say about nutrition with me being overweight.”* (Participant 2).

Understand the Importance of Health Promotion

All participants had at least basic knowledge concerning the health effects of nutrition, smoking and alcohol intake.

*“Because we know the effects of smoking on health, we, so we can, again, promote good health by having this knowledge”* (Participant 1).

Participants also understood the importance of addressing lifestyle risk factor behaviours when treating patients and seven of them talked about the importance of adopting a holistic approach when treating patients.

*“So I feel that as a physiotherapist, not just addressing a particular injury, but the overall impact of the patient’s lifestyle is very important…”* (Participant 13).

*“I think it's more of like treating the patient as a whole. I think that's actually an important factor because even if like for example, even if I'm giving them certain exercises, but their lifestyle pattern is the same, sometimes it will not have an impact.”* (Participant 10).

*“… if someone is not managing their, their high blood pressure and they're not managing their diabetes, because of the foods, then, you know, there's no point in us giving them exercises 'cause we still need to help them holistically because we've physiotherapy, you know…”* (Participant 1)

Feeling Confident When Discussing Certain Lifestyle Risk Factors

Some physiotherapists felt confident in discussing certain lifestyle behaviours with patients which came from either their knowledge or their own experiences.

*“I do feel confident (discussion nutrition), but it would be from experience rather than from again some formal education process that has been undertaken.” *(Participant 6).

*“When it comes to smoking, to be honest, I feel quite comfortable and confident as, because I have knowledge about ah, like how it kind of affects them (the patients).” *(Participant 11).

Furthermore, all participants felt confident in discussing PA levels compared to other lifestyle behaviours, because they had more knowledge about this health behaviour.

*“More confident than nutrition. Ahm… Definitely, you know, because well Physio is kind of physical activity profession.” *(Participant 5).

Despite this, it can be noticed that not all physiotherapists felt confident in discussing these lifestyle behaviours and some of them reported a lack of confidence when discussing nutrition and feeling* “Awkward”* (Participant 3) when discussing alcohol with their patients.

*“…possibly not feeling overly confident (discussing nutrition) about going into too much detail on it. You know, like I said at beginning people asking what's good food for this? What's good for that? I probably don't have the best answers to them, so I think that that is a…Something that I wouldn’t want to start going down a route where I couldn’t give a good quality answer.”* (Participant 16).

*“…I don't think, I think it is a bit more awkward (discussing alcohol), especially 'cause yeah. What is a unit? Most people don't really know what the unit is and just get quite difficult to quantify so.”* (Participant 4).

Barriers to health promotion

Appointments’ Short Duration

The short duration of the appointments was one of the main common barriers between participants in discussing lifestyle behaviours with patients.

*“We could do a whole healthy living session, but you know, with like 20-25 minutes and having to treat pain as the most, the biggest complaint, rather than something else, I think it is a barrier.”* (Participant 6).

 Physiotherapists’ Scope of Practice for Health Promotion

There were some inconsistencies among participants as to whether discussing different lifestyle risk factors was within their scope of practice. For some, it was not, but for others discussing these subjects should be part of a physiotherapist’s job leading to some confusion about what the exact role of a physiotherapist should be. This resulted in an internal conflict among some participants. As an example, Participant 1 believed that physiotherapists “…don’t have the expertise…” and discussing nutrition is out of a physiotherapist’s “…scope of practice.”. However, he believed that discussing this subject “…should really be a part of our job…”. 

*“I never go too far in (discussing nutrition), because it's not my scope and I know we should stay within our own scope.”* (Participant 7).

Participant 4 believed that discussing nutrition was part of the physiotherapists' scope of practice, but she had some difficulties raising this subject with her patients due to knowledge not being provided by the universities.

*“ … I think using the word “not in our scope” is a bit strong, 'cause it is (discussing nutrition)… but it's not something we get taught at university as such, ahh to focus on, in terms of our sort of physio advice.” *(Participant 4).

*“… if a patient isn’t bring it (nutrition) up, because we technically it's not our baseline training then should we bring it up? But it's just that slight confusion.” *(Participant 4).

Perception of Patient’s Reaction to Health Promotion Questions and Relevance of the Questions

Another common barrier between participants was believing that patients would respond negatively to questions about certain lifestyle behaviours. Discussions about these behaviours were often seen as a sensitive topic.

*“I would ask as a professional (about alcohol), but I think even for me to discuss it is sometimes sensitive because you never know… how the person woll, will react to that question. Ahm… I I think it's a sensitive subject, sure… I don't think it's comfortable as it discussing other things. Let's put it that way.”* (Participant 2).

Furthermore, participants stated that patients may also respond negatively to questions not expected from a physiotherapist. 

*“Probably a bit of of … uncomfortableness, like being uneasy about it (discussing smoking), not knowing how they will react…. 'cause we are physiotherapists and they don't expect that sort of advice from us.”* (Participant 14).

Some physiotherapists did not consider it relevant to ask about certain lifestyle behaviours even though they knew their importance to a person's health. As an example, Participant 1 understood that alcohol could impact people's relationships, their “organizational…skills, their mental health.” and alcohol could increase people’s weight. He believed that having this knowledge was important so physiotherapists “… can pass it onto individuals who… wasn’t aware that it was affecting their balance…” or their health. However, he normally does not talk about this subject with his patients.

*“I don't think it's relevant, sometimes you know someone complaining about back pain, how do I start talking about the alcohol?”* (Participant 1).

It was also observed that some participants consciously decide which lifestyle behaviours they would discuss with which patient. Normally, these discussions would only take place if these behaviours were relevant to the patient’s MSK condition that the physiotherapist was treating or if the physiotherapist suspected that the patient may have an alcohol problem.

*“Sometimes it can just be a perceptional problem when you've built a rapport with somebody you don't want to spoil that. If there is a general improvement. If if everything is fine, if they are otherwise healthy, or that's not something I want to get into (discussing smoking). Uhm, but yes, if I feel that this is something seriously impacting their condition, then only I will speak with them.” *(Participant 13)

*“Uhm, if I, it's not something that I would includes generally, UM, if I have any suspicions, and especially with patients that we have access to their medical records. If I have any suspicions of regarding their behavior at the time of the session, or if something that I seen on my notes I would ask if I have any basically, any flags. Uhm, other than that there's not something that I normally discuss (alcohol intake)” *(Participant 2).

Lack of Knowledge in Relation to Certain Lifestyle Behaviours Can Impact Their Counselling

Participants believed that their lack of knowledge concerning certain lifestyle risk factors, such as nutrition, alcohol and smoking was another barrier when counselling patients.

*“I kind of have quite limited knowledge so just don't wanna say something that patients says “oh physiotherapist told me to do this. That means I need to do this”, when the physio is not really know what she's talking about.”* (Participant 5). 

*“…I don't know enough (about alcohol) to be able to advise on that… I would feel really like… unconfident. Ahh I wouldn't know what to say, what to advise. I could sign post them absolutely, but if I was to give any education or advice around it, it would be very minimal.”* (Participant 14).

What became apparent from reviewing the results on “Barriers for Health Promotion” theme was that some participants identified the relevance of assessing lifestyle risk factors, but for all the reasons described above, this generally does not take place.

Knowledge and personal experiences

Physiotherapists’ Lifestyle Behaviours and Counselling

From analysing the interviews, there were multiple factors that influenced the participants’ counselling and guidance to their patients. Two participants found that their counselling was influenced by their knowledge, whereas other participants found that their guidance was directed by their own personal health decisions. However, for the majority, the participants found that their health decisions impacted positively on their counselling. Participant 2, would discuss certain lifestyle behaviours with patients, but would avoid discussing others. As an example, concerning smoking, she could share her personal experiences when counselling patients, but she avoided discussing nutrition because she felt she was not a good role model.

*“… professionally where I'm doing is based on my knowledge.”* (Participant 10).

*“Well, many times my patient have more physical activities than me, uh, I suppose that it's not my best period. Uh, in my life, but I I have a lot of knowledge about the exercises and how to ahhh to explain.” *(Participant 9).

*“It (not smoking) enables me to sort of… sell the advice and just have confidence. 'cause I feel like maybe if I smoked I wouldn't be as like fulfilling like forthcoming with it”* (Participant 4)

*“I think it impacts it, uh, positively… So, again, if we do find that somebody could struggle potentially from some sort of a gap in their nutrition, again my dietary habits could help me, help them.”* (Participant 6).

*“Well, it happened in the past ah patients that would tell me, “Oh, I've been smoking for a while and I don't think I'll be able to stop”. Uhm, efficient that I felt comfortable enough to do it (discuss smoking). I actually told them my case, I'm not an example for anybody, but if I can help, even just a little bit and to let them know that it is possible. It's not easy, but it is possible. I will let him know”* (Participant 2).

*“I think it's like I said to you, is like a a smoker telling you not to smoke. Uhm, so basically if you are. If you don't think in yourself as being a perfect example probably you would avoid having certain discussions with their patients just because you don't think you are doing as you are praising, basically.”* (Participant 2).

Internal Conflict

An internal conflict of participants is demonstrated in the quotes below. The benefits of adopting healthy lifestyle behaviours were identified, but not put into practice due to their preferences. 

*“I think knowledge-wise I have theoretical good knowledge (concerning nutrition and health), but practically I don't like follow it regularly”* (Participant 10).

*“What probably impacts on my food choice more than my own knowledge is what I like to eat.”* (Participant 16).

*“I'm not anxious about my life and my… health. It's something which we need to manage, but I I have not all the time my mind how to to protect my healthy.”* (Participant 9).

Solutions for health promotion

Improvements in Communication Skills, Discussions, Sharing Evidence Between Peers and Informative Leaflets

During the interviews, the participants presented some solutions for health promotion concerning all lifestyle behaviours analysed. The most common solutions were training to improve their communication skills, discussions and sharing evidence with their peers and having some informative leaflets they could share with their patients.

*“…the other thing is using lots of presentations which suppose… from the workplace or from a non-work they present… and also from the colleagues if they have got a good knowledge, I think that's a really good idea just to discuss with them, just to know that if if they had a similar patients or conditions what you're going to really advise them” *(Participant 15).

*“Let's say if you have some training on that (communication skills)… so you know, maybe sometimes I need to bring the conversation and… ask it in a way that we, it's not going to offend them or it's going to feel like totally natural…”* (Participant 17).

*“…if there is a like a sheet or a template which we can give every patient just to make our work easier and that's same, that means the same information is given out to everybody, every client, that is good. It's something they can pass on to their colleagues or friends as well to just increase the health in the community overall.”* (Participant 13).

## Discussion

This study aimed to explore the physiotherapists' perceptions of the influence of their health behaviours on their patients’ advice. The results showed that 13 participants reported their lifestyle behaviours impacted what they advise patients while for the other two, their counselling was based more on their knowledge. Of those 13, most of them believed their lifestyle behaviours impacted positively their counselling, while one participant stated avoiding discussing certain lifestyle behaviours that she was also struggling to follow. This is consistent with a cross-sectional study made by Vickers et al. [13], which showed that those primary care providers who had healthier behaviours were more likely to advocate them to patients and participants who were struggling to follow a specific health behaviour found counselling their patients on this challenging.

Providers who practice healthy lifestyle behaviours play an important role in helping patients also change their lifestyles to reduce their risk of chronic diseases [14] and those who share their personal experiences and serve as role models are perceived as being more motivating and credible [12,20]. Role modelling was seen as a driver for health promotion in our study, which is consistent with other quantitative and qualitative studies of physiotherapists [6,17]. In previous quantitative studies, patients, as well as physiotherapists, agreed that physiotherapists should act as role models concerning maintaining a healthy weight, practising regular PA, and avoiding smoking [11,17]. However, the majority of Black et al.’s [17] participants, who were physiotherapists, had healthy behaviours and the data collection was made through a questionnaire which led to self-reported data and social desirability bias. It would be interesting to understand, in future studies, the perceptions of physiotherapists who did not adopt these healthy behaviours concerning their role as role models. Practising what one preaches may have an important effect in helping patients adhere to healthier lifestyles [21]. Frank et al. and Lobelo [12,20] showed that patients had a higher motivation to improve their lifestyle if their health practitioner or other HCPs (physiotherapists not included in these quantitative studies) also engaged in healthy behaviours. For the majority of participants in our study, counselling of patients was largely influenced by their own health behaviours, and for some, being a role model was an important factor. Despite this, it is imperative to state that all participants must engage in evidence-based practice when treating patients and follow the code of conduct, performance and ethics from the Health and Care Professions Council (HCPC) [22].

All participants stated being healthy. However, despite none of them being smokers, four participants stated being overweight, another three reported they could/want to lose weight and one stated not being happy with her weight. According to Kyle et al. [10], there is a high prevalence of obesity in the UK among different HCPs, including physiotherapists. However, it is unknown the exact percentage of physiotherapists who were overweight in this study data was not collected to ascertain this [10]. Despite this limitation, the study’s results are important because it is known that obesity is a risk factor for the development of different NCDs and excess weight in HCPs can affect the effectiveness of health promotion messages delivered to their patients [3,10]. This point is highlighted in our study by one participant who stated being overweight and reported avoiding discussing nutrition with the patients. Concerning PA, seven participants of our study did not follow the recommended PA guidelines [23] and one of the main barriers reported was lack of time. According to a cross-sectional study by Lowe et al. [18], that assessed PA promotion in 522 UK physiotherapists’ routine practice, only 38% of them followed the recommended guidelines concerning PA levels. The reasons why most of these HCPs did not follow the guidelines were not explored in this study, which is a limitation, but their PA levels were not associated with their PA promotion activity [18]. These results were replicated in our study where all physiotherapists were confident in discussing PA with their patients despite seven of them not following the guidelines [23]. Concerning the participants of our study there is space for improvements in relation to their health. Another internal conflict was also seen between some participants’ knowledge and own health behaviours and this discordance between what they know and what they do may be related to a lack of self-efficacy and motivation, as well as costs and access to food/services/facilities that promote healthier behaviours [24]. It would be important that organisations that employ physiotherapists put in place strategies that can assist them to adopt healthier lifestyles, such as maintaining a healthy weight and improving their PA levels. This may not only improve their health, but it can also impact their health promotion counselling and, consequently, their patient’s health, through role modelling, as previously discussed [14].

Multiple barriers to health promotion were found in our study which included limited clinical time. This is consistent with other quantitative cross-sectional studies [9,24]. Furthermore, expecting higher contact time with patients may be unrealistic, so it would be important to change the perception of physiotherapists about their role in health promotion [6]. These HCPs have the advantage of having multiple appointments over a period of time with each patient when compared to other HCPs, which allows them to create a good rapport and trust between each other and provides them with the chance of multiple teachable moments that may potentially lead to patients’ behaviour change [25]. Furthermore, there was some confusion about what is the physiotherapists’ scope of practice which resonates with other quantitative and qualitative studies [6,9]. According to the Chartered Society of Physiotherapists (CSP), physiotherapists should provide brief interventions or advice concerning PA, tobacco use, poor diet and obesity as well as the risk of alcohol misuse [26]. Brief interventions or advice in these areas are known to be effective tools in preventative measures [26]. Other barriers to health promotion present in our study included the perceived patients’ reaction concerning being asked about certain lifestyle behaviours, as well as the relevance of those questions in relation to the patient’s current symptoms, which is comparable with other quantitative and qualitative publications [6,27]. However, a cross-sectional study by Black et al. [11] which aimed to understand patients’ opinions regarding physiotherapists discussing certain lifestyle behaviours showed that 91.3%, 73% and 51.3% agreed that these HCPs should discuss with them PA, maintaining a healthy weight and tobacco cessation, respectively. Nevertheless, this study only included 230 participants, so its results have limited generalizability [11]. Future studies with larger samples to understand how patients perceive the importance of behaviour change interventions provided by physiotherapists would be valuable.

Although some participants had confidence discussing certain lifestyle behaviours and all were confident in discussing PA with patients, there was a lack of knowledge and confidence in counselling about nutrition, smoking and alcohol which is in line with other quantitative studies of physiotherapists [9,24,27]. One of the reasons for this may be the fact that lifestyle behaviours are not systemically and consistently taught across international physiotherapy educational programs when compared to other competencies such as MSK and neurological areas [28,29]. The proportion of health promotion content in international physiotherapy curricula was not well studied and the available evidence only included the curricula from six English-speaking high-income countries, including the UK, so its results cannot be globally generalized [28]. According to this study, there was a small proportion of content in different physiotherapy educational programs related to primary and secondary disease prevention [28]. It would be important that physiotherapy accreditation bodies, academic institutions and professional bodies create universal standards and guidelines regarding health promotion, as well as assessment and management of lifestyle risk factors as clinical competencies for physiotherapists [28]. This would help the physiotherapists to prioritize the public’s health and well-being and holistically manage a patient’s presenting condition considering the individual's overall lifestyle-related behaviours and, on some occasions, their chronic diseases [5].

Strategies proposed by some of the participants to help them improve their health promotion skills were sharing leaflets with patients, peer discussions and sharing information and communication skills training. According to the results of a systematic review by Alexander et al. [30] that aimed to identify the types of educational content used by physiotherapists, the distribution of handouts/brochures was a common strategy that concurs with the results of our study. However, providing information alone, although it can be convenient, is an inadequate strategy to promote behaviour change, which is very complex [21,30], so other strategies (or a combination of strategies) may be warranted. More than communication skills training alone, it would be valuable to provide physiotherapists with the opportunity to undertake training on lifestyle behaviours (e.g., smoking cessation, alcohol misuse, basic nutrition counselling and sleep/stress management), patients’ readiness for change, motivational interviewing and knowledge about behaviour change models and theories so they can understand the reasons why patients sustain a positive or negative health behaviour [5,21,24]. This may also help break the “knowledge translation gap” where some HCPs understand the relationship between people's lifestyles and the development of NCDs, but they do not apply their knowledge by helping patients change their lifestyle-related health behaviours [29]. This point can be observed in some participants of our study where they understood how lifestyle behaviours could impact patients’ health, but they would not ask about or discuss these behaviours with them. Furthermore, employers need to make lifestyle learning materials accessible to HCPs [24]. Another strategy that could also be considered to help physiotherapists start the conversation concerning different health behaviours is the introduction of key questions on the assessment forms they need to fill out. For example, although Chow’s study was performed with physiotherapy students, it showed that when questions concerning smoking status and PA levels were added to the assessment form, they facilitated and prompted them to ask their patients about these behaviours [27].

If all the recommendations described above take place, then physiotherapists will have better knowledge and may adopt healthier lifestyle behaviours which may improve their health promotion messages to their patients and serve as role models which ultimately may have a positive public health impact. If the general population’s health could be improved through lifestyle changes, this might have a positive impact concerning the prevalence of NCDs and the mortality rates caused by these diseases in the UK and globally.

Strengths and limitations

One of the strengths of this study is the fact it addresses the main modifiable lifestyle risk behaviours that can lead to the development of NCDs. To the researchers’ knowledge is the first study that explores the drivers and barriers MSK physiotherapists (working in the UK) experience when counselling these behaviours as well as the impact of their lifestyle behaviours when advising patients. It also included physiotherapists originally from different countries, with different ages and years of experience that were living in different parts of the UK, which can impact the study’s transferability as each individual has their own perceptions and experiences. However, this study cannot be transferred to other physiotherapy specialities or other HCPs because the sample size only included MSK physiotherapists.

Despite our study including 15 interviews, it cannot be clear if data saturation was reached, so it is unknown if further insights would have emerged if the sample size had been greater [15,16]. All participants took part in the study voluntarily and some of them knew the main researcher which may indicate that all of them had an interest in the research topic and/or an interest in helping a work colleague and maybe felt confident in discussing health behaviours, leading to selection bias. This familiarity could establish a better rapport during some interviews, but at the same time could have impacted the participants’ answers which could have led to social desirability bias. Furthermore, on a few occasions, the main researcher needed to prompt the participants or use leading questions when they misunderstood certain open questions even after rephrasing, which could impact their answers, which is another limitation. Additionally, the fact participants work in the same company, can be another limitation as they will follow the company's culture and values. If similar studies are conducted in the future, it will be important that the researchers do not have this familiarity with their participants, to reduce the risk of influencing their answers. However, even if this occurs, social desirability bias may still take place. A larger sample size, including participants working in different organisations, should also be considered. Finally, to allow a richer exploration of this topic a mixed-method study where, through a questionnaire, researchers could identify participants with different lifestyle behaviours and select some of them, through purposive sampling, to perform an interview to understand if their views about their health behaviours and patient counselling are different should be considered.

## Conclusions

In this study, the majority of participants believed their lifestyle impacts their counselling to patients. Drivers for health promotion included being a role model, having some knowledge concerning certain lifestyle behaviours and understanding their importance in health. However, barriers to health promotion were also present. These included a lack of time and knowledge about certain lifestyle behaviours, some confusion if discussing some of these behaviours is included in their scope of practice, perception of patients’ reactions to certain questions and their relevance to the MSK condition the patient was experiencing.

The results of this study demonstrate the need for training physiotherapists about certain lifestyle behaviours, including smoking cessation, alcohol misuse and basic nutrition counselling which may improve their health promotion messages, potentially leading to patient behaviour change which ultimately may have a positive public health impact. Furthermore, physiotherapists should recognise that they have a crucial role in health promotion and brief discussions about different lifestyle behaviours are within their scope of practice.

## Appendices

**Table 3:** Guide for the semi-structured interview

Demographic information	Can you please tell me your age/height and number of years of experience? (all in separate questions);
	Where do you live at the moment?
	Where are you originally from?
	Currently, do you work only virtually, in a clinic or a mixture of both?
Present Medical conditions	How healthy do you think you are?
	How do you feel about your weight? Prompt: Do you think you have a healthy weight? Why do you think that?
	Currently, do you have any relevant health conditions?
	Do you mind if I ask you if you are taking any prescribed medication? If so, please tell me what is it.
Diet	How do you consider your current dietary habits are? If good/bad: What are your reasons to consider your dietary habits healthy/unhealthy?
	Do you currently follow a particular diet? (eg: vegetarian, vegan). Prompt: What is your reason to follow this particular diet?
	Do you think you follow the recommendations of eating 5 pieces of fruits and vegetables per day? Prompts: Why do you think this recommendation is important? Why is this important for you or what drivers/barriers do you have concerning following this recommendation?
	How often do you eat high-fat foods? Prompt: What are your reasons to eat this type of food xx times a week/month?
	How often do you eat processed foods? Prompt: What are your reasons to eat this type of food xx times a week/month?
	What is your knowledge concerning nutrition and health?
	Why it might be important to have some knowledge about this subject?
	Why might this knowledge impact food choices?
	Does the knowledge you have impact your food choices? If yes/no, why?
	Do you think physiotherapists should address nutrition during their sessions? Prompt: Why/Can you explore this a bit further?
	Do you tend to discuss/counsel nutrition to your patients? (if yes/no, ask to explore this a bit deeper. What are the barriers and drivers?)
	How do you feel when discussing nutrition with your patients? Prompt: do you feel confident/not confident? Why do you feel that way?
	What support do you think you need to help you address nutrition in your clinical practice?
	How do you think your dietary habits impact your counselling to patients? Prompt: Can you explore this a bit further?
Physical activity	How often do you practice any physical activity during the week?
	What drives you to practice PA? or What are your barriers to practice PA?
	What is your knowledge in relation to PA and health?
	Why do you think it is important to know the relation between PA and health?
	Why might be important for physiotherapists to address general advice in relation to PA levels, especially in sedentary patients?
	Do you tend to discuss PA levels with your patients (especially the ones who are more sedentary)? Prompt: What are your barriers and drivers to discuss this with them?
	How do you feel when discussing PA with your patients? Prompt: Why do you feel like that?
	What support do you think you need to help you addressing PA in your clinical practice?
	How do you think your PA levels impact your counselling to patients? Prompt: Can you explore this a bit further?
Smoking	If you do smoke, can you please tell me how many cigarettes you smoke in a day?
	What drives you to smoke (or not to smoke)?
	What is your knowledge in relation to smoking and health?
	Why do you think this knowledge is important?
	Why might be important for physiotherapists to address smoking in their clinical practice?
	Do you tend to discuss smoking with your patients? Prompt: What are your barriers and drivers?
	How do you feel when discussing smoking with your patients? Prompt: Why do you feel that way?
	What support do you think you need to help you addressing smoking in your clinical practice?
	How do you think your own choices in relation to smoking impact your counselling to patients? Prompt: Can you explore this a bit further?
Alcohol	If you do drink alcoholic beverages, can you please tell me how many you drink in a week?
	What drives you to drink (or not to drink)?
	What is your knowledge in relation to alcohol consumption and health?
	Why might having this knowledge is important? or How is your knowledge in relation to this topic?
	Why it might be important for physiotherapists to address alcohol in their clinical practice?
	Do you tend to discuss alcohol intake with your patients? Prompt: What are your barriers and drivers?
	How do you feel when discussing alcohol intake with your patients? Prompt: Why do you feel that way?
	What support do you think you need to help you addressing alcohol intake in your clinical practice?
	How do you think your drinking habits impact your counselling to patients? Prompt: Can you explore this a bit further?
